# Enhanced Pharmacy Service Delivery Through a Pharmacy Information System at a Medical Center: A Case Study

**DOI:** 10.7759/cureus.68865

**Published:** 2024-09-07

**Authors:** Sarah M Alanazi, Abdullah T Alanazi

**Affiliations:** 1 College of Public Health and Health Informatics, King Saud Bin Abdulaziz University for Health Sciences, Riyadh, SAU; 2 Health Informatics, King Abdullah International Medical Research Center, Riyadh, SAU; 3 Information Technology, King Abdulaziz Medical City, Riyadh, SAU; 4 Informatics, King Abdullah International Medical Research Center, Riyadh, SAU; 5 Health Informatics, King Abdulaziz Medical City, Riyadh, SAU

**Keywords:** errors, medication, pharmacy information system, pis, use of information

## Abstract

Introduction: A pharmacy information system (PIS) is a crucial tool for preventing errors and enhancing patient safety. This study aims to assess the influence of a PIS on workflow and medication errors (MEs) within the pharmacy department of a medical city in Saudi Arabia. The analysis encompasses an evaluation of the current workflow within pharmacy modules (in-patient, out-patient, and clinical pharmacy modules) and an exploration of associated challenges and inefficiencies.

Method: The study employed both qualitative and quantitative methodologies, incorporating structured interview techniques and the review of system records, such as logs, incident reports, and ME databases.

Results: Respondents recognized the benefits of the PIS, including the management of high-quality real-time data (26%), reduction of MEs (25%), acceleration of healthcare practices (23%), and absence of space-time constraints (14%). The study highlighted the role of PIS in addressing medication stock control and shortage issues, thereby improving overall medication availability. Furthermore, PIS significantly enhanced safety (93.3%), efficiency (80%), reliability, accuracy, ease of use (56.7%, 56.7%), and cost-effectiveness (53.7%). Following the implementation of the PIS, there was an increase in ME reporting, indicating improved error detection and management.

Conclusion: The research revealed an increase in ME reporting rates in hospitals after PIS installation, attributed to improved medication management processes resulting in higher detection rates. As healthcare facilities increasingly embrace electronic medical records, the understanding of patient care quality will be significantly impacted. Consequently, further research is warranted to explore the role of PIS in reporting, detecting, analyzing, and mitigating MEs, as well as their influence on the work environment to promote a secure and efficient patient journey.

## Introduction

The report "To Err is Human" by the Institute of Medicine underscored the imperative of establishing a safer healthcare system in response to the significant fatalities resulting from medical errors. The report emphasized the pressing need to diminish medical errors and advance patient safety, while also highlighting the criticality of refining flawed systems without assigning blame to healthcare professionals for inadvertent mistakes [1].

Electronic health record (EHR) systems function as a robust defense against errors, ensuring patient safety through the storage, management, and exchange of patient data for research, quality enhancement, and decision support. Data collected in care settings constitute an electronic medical record (EMR). EHRs consolidate EMRs from various locales to facilitate data exchange. EMRs facilitate timely access to patient data for appropriate management and early detection of complications and can monitor population health outcomes [2].

Numerous studies have demonstrated the impact of EMRs on patient safety and the healthcare system. Nonetheless, healthcare providers globally encounter challenges in the adoption and implementation of EMR due to insufficiencies in education, training, and acceptance. Integrating digital technology in healthcare facilities can enhance healthcare efficiency and patient care. In Saudi Arabia, each hospital devises its approach to implementing digital healthcare technologies based on its unique challenges. The implementation of EMRs in hospitals encounters barriers such as workflow inadequacies and issues related to inventory management. Opposition and challenges in meeting expectations and realizing desired benefits are common hurdles in the adoption of digital health technology [3].

In Saudi Arabia, the adoption of pharmacy informatics software in hospitals aims to heighten patient safety and healthcare quality. Evaluating the impact of EMR on pharmacy workflow and medication management procedures is crucial to maximize its benefits [4]. EMRs aid in various pharmacy tasks, streamlining clinical work and enhancing patient care. This research endeavor aims to augment the understanding and application of pharmacy informatics systems in a Saudi tertiary care hospital, to improve patient safety and workflow adherence while contributing to the field of health informatics [5].

In their 2006 paper, Garets and Davis delineated the disparity between EMRs and EHRs. An EMR encompasses a range of applications, including a clinical data repository, clinical decision support, controlled medical vocabulary, order entry, computerized provider order entry, pharmacy, and clinical documentation. It is a legal record produced in hospitals and ambulatory environments, serving as the primary source of data for the EHR [6]. Conversely, an EHR facilitates the effortless dissemination of medical information among stakeholders and ensures the seamless transfer of a patient's information across various care modalities. Stakeholders consist of patients, caregivers, medical professionals, employers, and government entities. In essence, an EMR lays the groundwork for a unified and accessible EHR.

In his 2023 review, Middleton exhaustively examined the available evidence on EMR implementation in all health settings in Australia. He aimed to identify current knowledge gaps and provide robust recommendations for future research on evaluation strategies. Conducting a comprehensive scoping literature review of primary, peer-reviewed, academic literature from 2012 to 2022, Middleton found glaring inconsistencies in the framework to evaluate EMR implementation in Australia. These discrepancies make it arduous to define the return on investment and its impacts on healthcare delivery. Despite the efforts to bolster the evidence base in Australia, Middleton argues that the current quality and quantity of available evidence falls short of catalyzing policy reform or shaping recommendations for future evaluation strategies due to the intricate nature of conducting research in this field. Notably, the evidence, mostly qualitative, remains constrained by research on various EMR systems in different settings and among different user groups. Nonetheless, the evidence strongly upholds EMR implementation, showcasing substantial benefits such as heightened efficiency, safety, and improved patient outcomes [6].

In their 2023 study, Setyadi et al. emphasized the critical importance of EMRs in reducing the burden, expenses, and errors for physicians. They unequivocally highlighted that easy access to patient data can significantly enhance clinical decision-making and patient safety, benefiting both medical professionals and patients. The study rigorously reviewed the literature published between 2020 and 2023 and focused on national and international research articles in English from sources such as Scopus, Science Direct, and PubMed. The robust findings strongly indicated that hospitals implementing EMRs demonstrated markedly improved service quality compared to those using paper medical records (PMR). EMRs unequivocally facilitate real-time access and updating of patient information, leading to significantly increased patient comfort and reduced waiting times, thereby profoundly enhancing healthcare efficiency [7].

This study aims to identify the impact of EMRs on medication errors (MEs) in a Saudi Arabian tertiary care facility, focusing on challenges and potential advantages in the pharmacy department. It addresses specific questions about workflow limitations, verification of drug prescriptions, difficulties in stock management, and real-world user perspectives.

## Materials and methods

This study was conducted at Riyadh Health Cluster, a medical city in Saudi Arabia that has introduced the EMR system after providing six months of extensive training and education to healthcare providers. The study used a combination of qualitative and quantitative methods to explore the challenges and opportunities associated with implementing EMR and integrating it with pharmacy informatics in the Saudi tertiary care hospital.

To collect data, structured interviews were carried out with key stakeholders (pharmacists, physicians, and nurses). The questions were adapted from previous research and were reviewed by three health informatics professionals with a pharmacy background, in collaboration with the pharmacy department, to ensure accuracy and clarity [8]. The questions focused on their attitudes toward the pharmacy information system (PIS) and how it impacted their workflow adherence, prescription verification, and stock management practices. Furthermore, the study involved document analysis by reviewing system records, such as logs, incident reports, and ME databases.

King Fahad Medical City - Riyadh Second Cluster issued approval 24-141. Information obtained in this study is strictly confidential and anonymity will be maintained.

## Results

The study encompassed interviews with thirty principal stakeholders, including 14 pharmacists (inclusive of in-patient, out-patient, and clinical pharmacists), nine nurses, and seven physicians. Among the respondents, 19 (63%) were male, 13 (43%) were aged 30-39, and half of them possessed bachelor's degrees (16 respondents, representing 53.3% of the total sample). One-third of the participants had six to 10 years of experience (10, 33.3%), followed by those with more than 15 years of experience (seven, 23%). Twenty-two (73%) of the respondents were involved in all three primary facets of the impact of the PIS, encompassing workflow adherence, prescription verification, and stock management processes. The demographic data of the respondents is explicated in Table 1.

**Table 1:** Demographics of the respondents PIS: pharmacy information system

Attribute	Levels	Values N (%)
Gender	Female	11 (36.7)
	Male	19 (63.3)
Age	20-29	10 (33.3)
	30-39	13 (43.3)
	40-49	2 (6.67)
	50-59	3 (10)
	60 and above	2 (6.67)
Specialty	In-patient pharmacists	5 (16.7)
	Out-patient pharmacists	5 (16.7)
	Clinical pharmacists	4 (13.3)
	Nurses	9 (30)
	Physicians	7 (23.3)
Education level	Bachelor's degree	16 (53.3)
	Master's degrees	10 (33.4)
	Doctoral degree	4 (13.3)
Experience (year)	1-5	9 (30)
	6-10	10 (33.3)
	11-15	3 (10)
	More than 15	7 (23.3)
Experience with PIS	PIS helps me in adherence workflow.	3 (10)
	PIS helps me in prescription verification.	5 (16.7)
	PIS helps me in stock management.	0 (0)
	PIS helps me in all the above tasks.	22 (73.3)

In terms of the benefits of utilizing a PIS, the data indicate that respondents attributed various advantages to the system. These include the effective management of high-quality real-time data (26, 87%), the reduction of MEs (25, 83%), the acceleration of processes in healthcare practices (23, 77%), and the absence of space-time constraints (14, 47%). However, PIS (EMR) users have raised concerns about the time consumption, with each prescription taking approximately 10 minutes to process, resulting in delays. The vast majority of respondents (26, 86.7%) recognized the potential of the PIS to streamline adherence workflow, enhance verification accuracy, and optimize stock management within the pharmacy system. The respondents emphasized the important role of the PIS in improving safety (28, 93%), efficiency (24, 80%), reliability, accuracy, and user-friendliness (17, 57% each), as well as cost-effectiveness (16, 54%).

Key concerns about the PIS included lack of flexibility, absence of regulatory standards, insufficient training, and limited understanding of the system. There are deficiencies in PIS training (24, 80%), highlighting the critical need for training on the advantages and limitations of PIS applications (24, 80%), technical methods (24, 80%), clinical use of PIS applications (23, 77%), and effective operation of PIS (15, 50%).

The participants strongly advocate for a collaborative effort among PIS vendors, hospitals, and international organizations to ensure that the full potential benefits of the PISs are realized. For the chart analysis, the number of reported MEs in different departments of pharmacy is presented in Table 2.

**Table 2:** Number of reported MEs in different departments of pharmacy before and after PIS ME: medication error, PIS: pharmacy information system

	Pharmacy supply N (%)	In-patient pharmacy N (%)	Clinical pharmacy N (%)	Ambulatory pharmacy N (%)	Total
One year before PIS	6 (0.09)	5768 (88.6)	5 (0.08)	733 (11.26)	6512
One year after PIS	8124 (59.6)	1420 (10.4)	46 (0.34)	4048 (29.7)	13638

The number of reported MEs in different departments reported by nurses is presented in Table 3.

**Table 3:** Number of reported MEs in different departments reported by nurses before and after PIS ME: medication error, PIS: pharmacy information system

	WSH nursing N (%)	Surgical nursing N (%)	Rehabilitation nursing N (%)	Medical nursing N (%)	Children nursing N (%)	Total
One year before PIS	138 (25.2)	111 (20.26)	71 (13)	199 (36.4)	29 (5.3)	548
One year after PIS	94 (20.4)	66 (14.3)	46 (10)	91 (19.8)	163 (25.4)	460

The percentage of MEs reported before and after PIS in all hospitals within the medical city is detailed in Table 4.

**Table 4:** Percent of MEs reported before and after PIS in all hospitals within the medical city ME: medication error, PIS: pharmacy information system

Type of MEs	PIS	Hospital 1	Hospital 2	Hospital 3	Hospital 4
Administration errors (%)	Before	67%	21%	9%	3%
	After	37%	22%	25%	16%
Prescribing errors (%)	Before	60%	18%	17%	5%
	After	38%	37%	17%	8%
Documentation errors (%)	Before	23%	66%	8%	3%
	After	7%	92%	1%	0%
Monitoring errors (%)	Before	14%	29%	43%	14%
	After	15%	34%	39%	12%
Preparation errors (%)	Before	81%	10%	7%	2%
	After	47%	38%	9%	6%
Procurement errors (%)	Before	57%	29%	0	14%
	After	53%	36%	5%	6%
All types of MEs (%)	Before	57%	18%	17%	8%
	After	41%	39%	13%	7%

## Discussion

The use of health technology in healthcare systems has been very supportive and helpful in organizing patient information and promoting evidence-based practice for high-quality patient care, ensuring access to safe care (20). Pharmacists are the most active users of the PIS (14, 46.7%), followed by nurses (nine, 30%) and physicians (seven, 23%). Meanwhile, inpatient and outpatient pharmacists are almost equally using the PIS (five, 16.7%).

Most of the respondents favor the use of the PIS as they expect themselves to be familiar with it, considering its useful application in the healthcare field. This aligns with a similar study by Jabali and Abdulla (2023), which found a positive perception among Saudi Arabian respondents about PIS use [9]. There is also a strong belief that the implementation of the PIS can play a role in controlling and reducing MEs, improving medication safety and care, facilitating prescription verification, aiding in stock management, and serving as a quality control tool. It also enables the respondents to be more efficient and reduces the burden on nurses. This aligns with the findings of a similar study by Manca (2015), which found that EMRs enhance the quality of care, patient outcomes, and safety by improving management, minimizing MEs, reducing redundant investigations, and improving communication between patients and their healthcare providers [10]. Overall, it is clear that there is no fear of PIS replacing practitioners; instead, there is a belief that it can bring positive change to the work environment and improve patient care. This belief is consistent with the findings of a study by Tabche et al. (2023), which mentioned that the use of EHRs assists doctors in recording patient details, including medications and treatment procedures, as well as their outcomes [11].

The study indicates that respondents believe that PIS has several advantages. First, it can effectively manage high-quality data in real time (26, 87%). Second, it helps in reducing MEs (25, 83%). Third, it speeds up processes in healthcare practices (23, 77%), and lastly, it has no space-time constraints (14, 47%). However, PIS users believe that the system consumes too much time, with an average of 10 minutes per prescription, leading to delays in processing. This issue can be addressed through efficient training and by minimizing processing clicks, for example, by combining two steps into one. The study also reveals that 26 (87%) of respondents understand the capabilities of PIS in enhancing workflow adherence, improving verification accuracy, and optimizing stock management within the pharmacy system. Given this understanding, respondents are willing to undergo training to learn the functions of the PIS. In addition, 25 (83.8%) of the respondents recognize that the PIS can be a good solution to improve overall medication availability, which is a serious concern from a medication stock control and shortage perspective, as supported by several studies [12,13].

The study shows that the PIS plays a significant role in improving safety (28, 93.3%), efficiency (24, 80%), reliability and accuracy, ease of use (17, 56.7%), and cost-effectiveness (16, 53.7%). However, a major concern raised by respondents is the lack of sufficient flexibility, regulatory standards, training, and understanding of the system (21, 70%). There are weaknesses in PIS training, with a need for training on the advantages and limitations of PIS applications (24, 80%), technical methods (24, 80%), clinical use of PIS applications (23, 76.7%), and how to use PIS effectively (15, 50%). The participants believe that there should be shared responsibility between PIS companies, hospitals, and international organizations to maximize the benefits of the PIS.

The study also presents data on MEs in the pharmacy department. Before the implementation of the PIS, the inpatient pharmacy had the highest number of MEs (5,768) in comparison to other pharmacy departments. After implementing PIS, the number of MEs in the inpatient pharmacy was reduced to 1,420, and the detection of MEs in other pharmacy areas, such as ambulatory care, increased from 733 to 4,048. The overall analysis revealed a non-significant p-value (P = 0.2, in which p < 0.05 is considered statistically significant), suggesting that the number of errors detected by the EMR is higher and can be managed effectively to prevent errors.

The study also provides statistics on the types of MEs in tertiary care hospitals. It explains the improved awareness and better detection and reporting of MEs after the implementation of the PIS. An increase in MEs was observed after implementing PIS in one study, but in our analysis, the rise in the detection of MEs following enhanced medication management processes indicates significant improvement. This can lead to proper analysis and management by performing root cause analysis (RCA). The study also details how the implementation of the PIS in nursing areas led to a reduction in MEs from 574 errors to 462. However, this reduction did not reach a significant level, as the PIS system is still on the learning curve within the organization (P = 0.5).

Limitations

The case study method provides a detailed and comprehensive understanding of PIS implementation in a medical facility. However, it's important to note that these findings may not apply to other contexts. Therefore, caution should be exercised when generalizing the results, and further research is necessary. It is also worth considering that conducting case studies can be a time-consuming process due to the intensive and lengthy data collection involved.

## Conclusions

The PIS not only helps pharmacists manage drug supply and organization but also plays a critical role in reducing MEs, improving patient safety, tracking drug usage, and monitoring costs. However, this study has shown that hospitals may experience an increase in MEs after implementing the PIS. This rise in reported errors can be attributed to improved medication management processes, leading to the identification of more errors. As hospitals increasingly adopt electronic medical records, it is clear that this will have a significant impact on patient care quality. Further research is essential to thoroughly assess the role of the PIS in reporting, detecting, analyzing, and reducing medication errors, as well as its impact on the work environment to enhance patient safety and operational efficiency.

## References

[1] Institute of Medicine (US) Committee on Quality of Health Care in America (2000). To err is human: building a safer health system.

[2] Flatow VH, Ibragimova N, Divino CM, Eshak DS, Twohig BC, Bassily-Marcus AM, Kohli-Seth R (2015). Quality outcomes in the surgical intensive care unit after electronic health record implementation. Appl Clin Inform.

[3] Darwish A, Hassanien A, Elhoseny Elhoseny (2019). The impact of the hybrid platform of internet of things and cloud. J Ambient Intell Human Comput.

[4] Siddiqui M, Majeed F (2023). A comprehensive review of current developments and future outlook pertaining to electronic medical records in the context of saudi arabia, aimed at enhancing the healthcare system. Preprints.

[5] Chapuis C, Roustit M, Bal G (2010). Automated drug dispensing system reduces medication errors in an intensive care setting. Crit Care Med.

[6] Garets D, Davis M (2006). Electronic medical records vs. electronic health records: yes, there is a difference. a HIMSS Analytics White Paper. DocPlayer.

[7] Michelle Middleton (2023). Evaluating the impact of EMR implementation in the Australian health system: a scoping literature review. August 06.

[8] El.Mahalli A, El-Khafif SH, Yamani W (2016). Assessment of pharmacy information system performance in three hospitals in Eastern Province, Saudi Arabia. Perspect Health Inf Manag.

[9] Setyadi D, Nadjib M (2023). The effect of electronic medical records on service quality and patient satisfaction: a literature review. J Res Soc Sci Econ Manag.

[10] Jabali AK, Abdulla FA (2023). Electronic health records perception among three healthcare providers specialties in Saudi Arabia: a cross-sectional study. Healthc Technol Lett.

[11] Manca DP (2015). Do electronic medical records improve quality of care? Yes. Can Fam Physician.

[12] Tabche C, Raheem M, Alolaqi A, Rawaf S (2023). Effect of electronic health records on doctor-patient relationship in Arabian gulf countries: a systematic review. Front Digit Health.

[13] Shermock SB, Shermock KM, Schepel LL (2023). Closed-loop medication management with an electronic health record system in U.S. and Finnish hospitals. Int J Environ Res Public Health.

